# Expression Profiles of Alkaloid-Related Genes across the Organs of Narrow-Leafed Lupin (*Lupinus angustifolius* L.) and in Response to Anthracnose Infection

**DOI:** 10.3390/ijms22052676

**Published:** 2021-03-06

**Authors:** Katarzyna Czepiel, Paweł Krajewski, Paulina Wilczura, Patrycja Bielecka, Wojciech Święcicki, Magdalena Kroc

**Affiliations:** 1 Legume Genomics Team, Institute of Plant Genetics, Polish Academy of Sciences, Strzeszyńska 34, 60-479 Poznań, Poland; kcze@igr.poznan.pl (K.C.); mwil@igr.poznan.pl (P.W.); bieleckapat@gmail.com (P.B.); wswi@igr.poznan.pl (W.Ś.); 2 Biometry and Bioinformatics Team, Institute of Plant Genetics, Polish Academy of Sciences, Strzeszyńska 34, 60-479 Poznań, Poland; pkra@igr.poznan.pl

**Keywords:** narrow-leafed lupin, alkaloids, transcription factor *RAP2-7*, plant-pathogen interaction, expression profiles, *Colletotrichum lupini*, alkaloid-related genes

## Abstract

The main restraint obstructing the wider adoption of lupins as protein crops is the presence of bitter and toxic quinolizidine alkaloids (QAs), whose contents might increase under exposure to stressful environmental conditions. A poor understanding of how QAs accumulate hinders the breeding of sweet varieties. Here, we characterize the expression profiles of QA-related genes, along with the alkaloid content, in various organs of sweet and bitter narrow-leafed lupin (NLL, *Lupinus angustifolius* L.). Special attention is paid to the *RAP2-7* transcription factor, a candidate regulator of the QA pathway. We demonstrate the upregulation of *RAP2-7* and other QA-related genes, across the aerial organs of a bitter cultivar and the significant correlations between their expression levels, thus supporting the role of *RAP2-7* as an important regulatory gene in NLL. Moreover, we showed that the initial steps of QA synthesis might occur independently in all aerial plant organs sharing common regulatory mechanisms. Nonetheless, other regulatory steps might be involved in *RAP2-7*-triggered QA accumulation, given its expression pattern in leaves. Finally, the examination of QA-related gene expression in plants infected with *Colletotrichum lupini* evidenced no connection between QA synthesis and anthracnose resistance, in contrast to the important role of polyamines during plant–pathogen interactions.

## 1. Introduction

Industrial and consumer interest in lupins grew recently due to their wide range of agricultural and health benefits, as well as their significant contribution toward achieving sustainable farming [1,2]. Lupin seeds are increasingly considered a valuable protein source in terms of both the human diet and as animal fodder, due to their high protein content [1]. Like other legumes, lupins fix atmospheric nitrogen, thus supplying the soil with bioavailable nitrogen necessary for the cultivation of other crops, and allowing for reduction of mineral fertilizer use [3,4]. The main restraint obstructing the wider adoption of lupin as a crop results from the presence of alkaloids, which although considered to provide substantial chemical defense against herbivores and pathogens, are also considered to be antinutritional factors in terms of both food and feed, due to their bitter taste, simultaneous toxicity, and tendency to accumulate in lupin seeds [5,6]. The alkaloids occurring in lupins mostly belong to the group of quinolizidine alkaloids (QAs), which are lysine-derived molecules distributed mainly in the Leguminosae family [7,8].

Narrow-leafed lupin (NLL, *Lupinus angustifolius* L.) is an important legume crop with a relatively short breeding history [9]. During the process of NLL domestication, a natural, recessive mutant allele, *iucundus*, underpinning a reduced alkaloid content was discovered, which was since incorporated into most *L. angustifolius* cultivars, along with other domestication genes [9,10]. It is evident that breeding efforts might reduce the alkaloid content below the established safety threshold (0.02% of seed dry weight) [11]. However, success in the effective and sustained reduction of seed alkaloid content relies on the detailed clarification of the molecular mechanisms involved in the QA synthesis pathway, as well as its underlying regulation.

Whereas the molecular machinery underlying alkaloid accumulation is quite well-resolved in other plant species (e.g., *Catharanthus roseus* and *Nicotiana tabacum*), researchers working with lupins are still struggling to paint a general picture of QA synthesis and pathway regulation [8,12,13,14,15,16,17]. Various theories about the sites of QA synthesis in lupins emerged over the years [12,18,19,20], coalescing into a consensus that the vast majority of QA synthesis takes place in green aerial tissues [21], with a small contribution from the roots [14]. The existing knowledge about leaf chloroplasts, as a site of QA biosynthesis [22], is enriched by recognition of the input of other lupin organs, especially pods [14,23] and stems [24]. The very first steps of QA synthesis seem to be relatively well understood. At the beginning, inside plant chloroplasts, lysine is decarboxylated into cadaverine polyamine [15,22,25], which is further converted into 1-piperideine [16]. These two reactions are carried out by lysine decarboxylase (LDC) [15] and copper amine oxidase (LaCAO) [16] enzymes, respectively. Another enzyme, namely the mitochondria (−)-13α-hydroxymultiflorine/(+)-13α-hydroxylupanine O-tigloyltransferase (HMT/HLT), is involved in the formation of ester-type alkaloids, which are assumed to be a form of alkaloid transport and storage [13,20,26]. Additionally, acyltransferase enzyme (LaAT) is described as possibly being involved in the formation of polyamine conjugates. However, its specific function needs further investigation [8]. We recently demonstrated that the APETALA2/ethylene response RAP2-7 transcription factor (TF) likely plays a crucial role in the regulation of alkaloid accumulation in NLL [27]. The *RAP2-7* gene is found to co-segregate with the *iucundus* locus, conferring low seed alkaloid content, and is located within a region with major QTLs that affect the QA composition. The contribution of this genomic region to QA biosynthesis is further supported by the 4-hydroxy-tetrahydrodipicolinate synthase (*DHDPS*) gene that is possibly involved in L-lysine biosynthesis, which is found to be closely linked with the *iucundus* locus [27]. Furthermore, a gene-targeted marker developed based on the *RAP2-7* point mutation showed 100% marker–trait association (199 *iucundus*/*Iucundus* accessions comprising domesticated and wild germplasm) [28]. Last, but not least, we confirmed that the *RAP2-7* expression was much higher in the leaves of high-alkaloid *Iucundus* vs. low-alkaloid *iucundus* accessions, in a previous transcriptome-derived investigation [27]. Nonetheless, until now, *RAP2-7* expression profiles were not yet investigated in other NLL organs.

Lupin anthracnose, caused by the fungal pathogen *Colletotrichum lupini*, is one of the most devastating lupin diseases and leads to major agricultural losses [29]. Plants exposed to various stress-inducing agents produce a wide range of different secondary metabolites, many of which play an important role in the response to biotic stresses [5,30]. However, the detailed involvement of QAs in response to anthracnose infection remains obscure. It was shown [31] that a high alkaloid content in *Lupinus arboreus* correlated with lesser lesions caused by *Colletotrichum* sp. pathogens. In contrast, an evaluation of secondary metabolite profiles of NLL cultivars exposed to *Colletotrichum lupini* revealed that alkaloid accumulation was not induced by the infection; however, the correlation between plant resistance and QA accumulation is not yet investigated [32]. At present, only limited sources of resistance against anthracnose are available in breeding programs [29]. Therefore, an improved understanding of the relationship between plant exposure to *Colletotrichum lupini* and QA synthesis is crucial in considering them as another line of defense against this pathogen in lupins. Moreover, determination of whether the infection of resistant accessions is accompanied by an increase in seed QA content is also pivotal from the perspective of food security.

In the current study, we explored the association between the expression profiles of QA genes and alkaloid content/composition, as estimated in selected organs of NLL, to further discern the significance of the tested organs in QA biosynthesis. We analyzed organ-specific expression patterns of QA structural and candidate genes, with special attention paid to the *iucundus* candidate, in the form of *RAP2-7*, to further explain its role in the regulation of QA biosynthesis. Moreover, we also examined the transcriptional changes of QA genes in NLL plants infected with *Colletotrichum lupini*, to investigate the possible role of lysine-based alkaloids as a secondary line of plant defense against infection by this fungus.

## 2. Results

The expression profiles (quantitative PCR assay, qPCR) of seven genes involved in QA biosynthesis and accumulation (Table 1) and their relation to alkaloid content (gas chromatography assay, GC) were analyzed in the organs of two NLL cultivars. We chose cultivars with contrasting phenotypes; namely, Oskar (high-alkaloid, bitter) and Regent (low-alkaloid, sweet). A total of 13 types of samples representing plant organs in different phenological phases were analyzed per cultivar (Table 2, Tables S1 and S2). Moreover, we determined the expression profiles of QA-related genes (Table 1) in the seedlings of eight NLL cultivars subjected to *Colletotrichum lupini* infection.

### 2.1. QA Gene Expression in Different Organs of Sweet and Bitter Lupin Cultivars

Through ANOVA, a statistically significant interaction between the effects of plant organ and cultivars (of sweet and bitter phenotypes; *p* < 0.01) was identified in the case of the expression of cinnamoyl-reductase 2 (*CCR*), *HMT/HLT*, *LaAT*, *LaCAO*, *LDC*, and *RAP2*-7, while a significant interaction was not observed for *DHDPS* (Table 3). The mean expression of *HMT/HLT* appeared to be similar in both cultivars (Table 3). Log_2_-transformed mean values of gene expression in individual plant organs and their standard errors (SEs) are given in Table S3.

**Figure 1:**
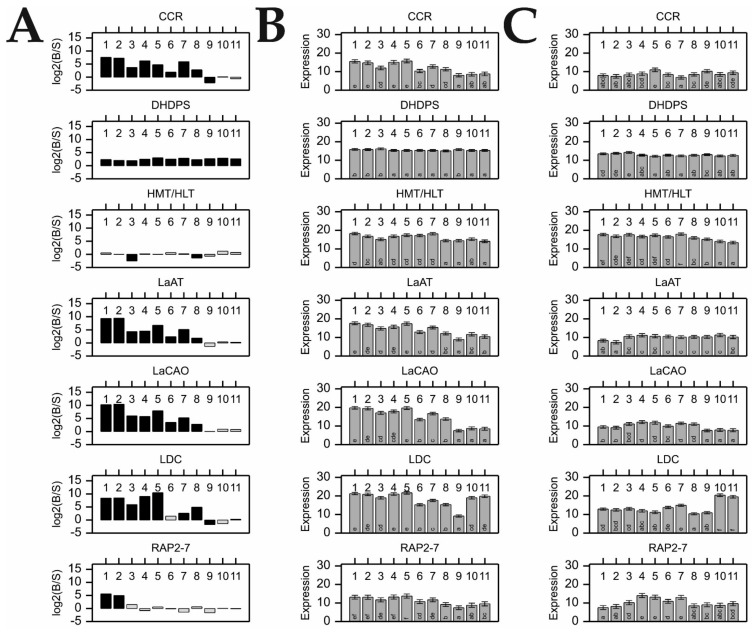
Alkaloid (QA) gene expression in various organs of two narrow-leafed lupin cultivars—Oskar (high-alkaloid, bitter) and Regent (low-alkaloid, sweet). Gene expression is based on log_2_-transformed CP_NORM values. Numbers from 1 to 11 refer to the following organs. 1. Leaves I; 2. Leaves II; 3. Leaves III; 4. Main stem; 5. Lateral stems; 6. Flowers; 7. Young pods; 8. Green pods; 9. Green seeds; 10. Main root; and 11. Lateral roots. Mean values and standard errors are shown. For a detailed list of gene names and organs, see Table 1 and Table 2, respectively: (**A**) Fold-change (FC) of QA gene expression in the organs of bitter vs. sweet cultivar. The bars above the x-axis indicate higher expression in the bitter cultivar, while those below the x-axis represent higher expression in sweet cultivar. Black bars indicate statistically significant FC expression (*p* < 0.05). FC of expression is shown in log scale; (**B**) QA gene expression values in different organs of Oskar; and (**C**) QA gene expression values in different organs of Regent. Lettering on the bars indicate groups found using Fisher’s protected Least Significant Difference (LSD) test at *p* < 0.05.

We observed significant differences between cultivars in the expression level (i.e., fold change; FC) of investigated genes in particular organs (Figure 1A). The expression of most QA-related genes was much higher in the organs of the bitter cultivar. Taking that into account, the precise patterns of expression could be roughly divided into four groups. The first group was represented by *RAP2-7*, for which expression was significantly higher only in Oskar’s leaves I and II. The second group comprised four genes, namely *CCR*, *LaAT*, *LaCAO*, and *LDC*, which displayed the highest FC in the leaves, stems, and young pods of Oskar (occasionally other organs as well), whereas their expression did not differ significantly in roots. *HMT/HLT* represented a third pattern, the expression of which was comparable in the corresponding organs of the bitter and sweet phenotypes, with minor exceptions recorded for leaves III and green pods, where higher expression was recorded for Regent. The fourth expression pattern was observed for *DHDPS*, which was uniformly expressed at high levels in all organs of Oskar.

Closer inspection of gene expression in particular organs showed that all genes except for *HMT/HLT*, were more highly expressed in bitter leaves I and II; while all genes except for *RAP2-7* and *HMT/HLT* were more highly expressed in bitter leaves III, stems, and young and green pods (Figure 1A). In the case of the flowers of Oskar, the expression of four genes (*CCR*, *LaAT*, *LaCAO*, and *DHDPS*) was higher, as compared to their sweet counterparts, although the recorded FC was lower than for the aforementioned organs. Finally, there was almost no difference in the expression of QA-related genes in the roots and green seeds of Oskar, as compared to Regent (Figure 1A).

Differences between QA-related gene expression in various types of certain organs (i.e., parts of the root/stem structure) and organs at different developmental stages (i.e., leaves in successive stages of plant growth or maturing fruit) were individually analyzed for both cultivars (Figure 1B,C). Apart from the *DHDPS* gene showing quite uniform expression in all organs, several organ-specific expression patterns were observed in the bitter cultivar. For the investigated genes, expression was the highest in the green aerial organs of the plant, starting with leaves I and lateral stems, followed by leaves II and III, the main stem, and young pods, emerging in varying order for different genes (Figure 1B). Compared with those organs, the expression in flowers was visibly lower. Our results also showed that the expression level in leaves decreased with progressing time and plant development, with the highest expression occurring during plant flowering (leaves I) and the lowest after fruit ripening (leaves III). For most analyzed genes, with the clear exception of *LDC*, the lowest expression levels were observed in the roots and green seeds. Comparison of different parts of the root/stem structure showed no statistically significant differences between them. In the case of the sweet cultivar, expression of the tested genes was much more uniform within the organs, with the exception of significantly higher *LDC* expression in roots (Figure 1C).

Comparison of the organ-specific expression patterns obtained individually for the bitter and sweet cultivars (Figure 1B,C) distinguished two specific outcomes. First, the expression of *RAP2-7* across the organs of Oskar displayed a pattern of upregulation comparable to other QA genes in this cultivar, which was the highest in leaves I and lateral stem, followed by the remaining green aerial organs (Figure 1B). In the sweet cultivar, *RAP2-7* expression level was comparable to that of the bitter cultivar in all organs; except for leaves I and II, where the expression seemed to be downregulated in Regent (Figure 1C). As a result, a lack of differential *RAP2-7* expression was detected between cultivars, with the exception of leaves I and II (Figure 1A). Second, roots of both sweet and bitter cultivars were characterized as having the highest expression of *LDC* in comparison to the other QA-related genes in this organ (Figure 1B,C).

### 2.2. Correlations of QA Gene Expression Levels

**Figure 2:**
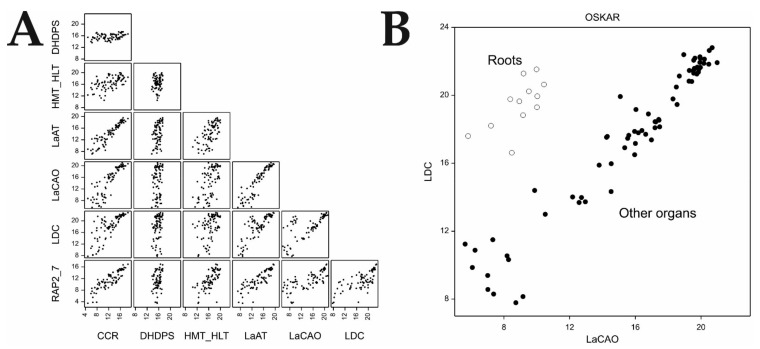
Graphical representation of the relationships between QA gene expression values observed in individual samples (log_2_-transformed CP_NORM values) in the bitter narrow-leafed lupin cultivar Oskar (*n* = 81): (**A**) Scatterplots of expression values in different organs; and (**B**) annotated scatterplot of *LDC* vs. *LaCAO* expression values. Results obtained for ‘root’ samples (empty circles) clustered separately from the remaining organs (black dots). Full names of genes are listed in Table 1.

In the sweet cultivar, correlations between the expression of QA genes were either significant but weak or not significant (*p* > 0.01), possibly due to low expression values (Table S4). In the case of the bitter cultivar, a strong positive correlation (*p* < 0.01) was observed between all tested genes; only *DHDPS* showed a weak correlation with other genes (Table 4, Figure 2A). The strongest mutual correlation was revealed for *CCR, LaAT, LaCAO, LDC,* and *RAP2-7* gene expression. The relationship of *LDC* expression with other QA genes did not always follow a linear pattern. Closer inspection revealed that this was caused by the expression results detected for root samples, constituting a separate cluster, which was particularly evident in relation to the *LaCAO* and *LDC* expression (Figure 2B). Exclusion of roots from the analysis noticeably increased the correlation coefficients between the expression of *LDC* and other genes (Table S5).

### 2.3. Determination of Quantitative and Qualitative QA Content in Plant Organs

**Figure 3:**
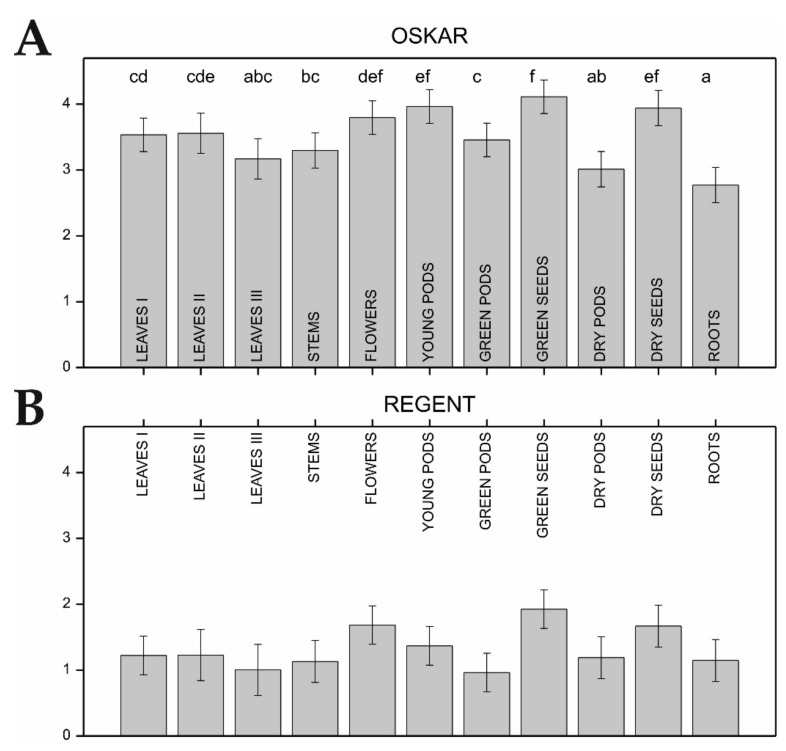
Total alkaloid content (TAC) in particular organs of narrow-leafed lupin measured by gas chromatography (GC) assay—(**A**) bitter cultivar Oskar; and (**B**) sweet cultivar Regent. Mean values and standard errors are depicted. In the bitter cultivar, groups of similar organs, found using Fisher’s protected LSD test (*p* < 0.05) are indicated by the same lowercase letter. No significant differences in QA content across organs were found for the sweet cultivar. TAC values are shown as log_10_-transformed values.

Total alkaloid content (TAC) assessed for all organs of the bitter cultivar largely exceeded the corresponding values measured in the sweet cultivar (Figure 3). The observed differences in TAC across the organs of Oskar were significant (*p* < 0.001, Figure 3A), although the transitions were not clear-cut. Further exploration of the identified TAC differences, supported by Fisher’s protected Least Significant Difference (LSD) test (*p* < 0.05), showed that the highest TAC was detected in the green and dry seeds, young pods, and flowers (Figure 3A) of the Oskar cultivar. On the other hand, the lowest TAC was found in the roots and dry pods of Oskar. Moderate TAC values were observed in the remaining organs. No significant differences were found between the TAC values for the different Regent organs (Figure 3B).

The ANOVA analysis found no statistically significant interaction between the effects of plant organ and cultivar on qualitative alkaloid composition of different organs of the studied cultivars (*p* > 0.01, Table 5). Differences between plant organs were not significant, with the exception of 13-benzoyloxylupanine. However, we found that the relative abundances of ten alkaloids were significantly different in the tested cultivars (*p* < 0.01, Table 5). The mean values of the relative abundances of individual alkaloids across the organs of the bitter and sweet cultivars are summarized in Table S6.

### 2.4. QA Gene Expression Changes in Response to Colletotrichum lupini Infection in Resistant and Susceptible NLL Cultivars

The ANOVA evaluating the differences in QA gene expression in two groups of NLL cultivars with different resistance levels to *Colletotrichum lupini* infection at different time-points of infection found no significant interactions between both variables (*p* < 0.01, Table 6). However, we found that the expression of four genes (*CCR*, *DHDPS*, *HMT/HLT*, and *LaCAO*) changed significantly at different infection time-points, while the expression of *DHDPS*, *LaAT*, and *LDC* was different between the tested groups of resistant and susceptible cultivars (*p* < 0.01, Table 6).

**Figure 4:**
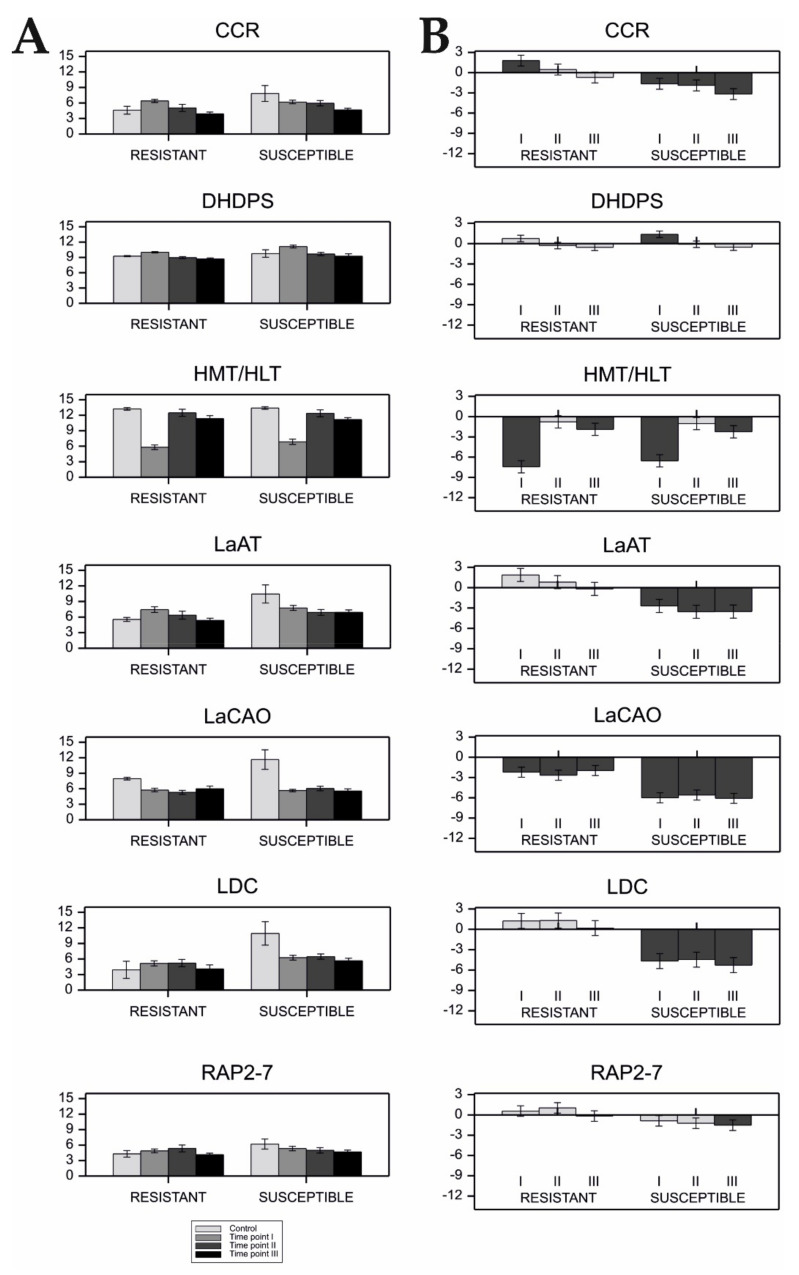
The expression pattern of alkaloid (QA) genes measured during *Colletotrichum lupini* infection in two groups of narrow-leafed lupin cultivars with different infection resistance levels. Plants mildly affected by the disease are referred to as ‘resistant’ (four cultivars), while plants severely affected by the disease are referred to as ‘susceptible’ (four cultivars). Full names of genes are listed in Table 1. (**A**) QA gene expression measured in control plants and at three time-points during *Colletotrichum lupini* infection (48 h, 72 h, and 120 h after inoculation). Log_2_-transformed mean values and standard errors are depicted. (**B**) FC values for QA gene expression at consecutive time-points of infection (I–III) in comparison to the control. Dark grey bars indicate statistically different expression (*p* < 0.05). Bars above the x-axis represent increased expression and those below the x-axis represent decreased expression after infection. FC of expression is shown in log_2_ scale.

In the case of susceptible cultivars, a significant increase in gene expression after infection was observed only for *DHDPS* (time point I), whereas the expression of the remaining genes, in most cases, decreased (Figure 4). In the resistant cultivars, the expression of *LDC*, *LaAT*, *RAP2-7*, and *DHDPS* genes did not change significantly after infection (Figure 4). On the other hand, the expression of *CCR* rapidly increased at time point I, returning to its previous level afterwards. The expression patterns of *HMT/HLT* and *LaCAO* were similar in susceptible and resistant plants after infection. In both groups of cultivars, *HMT/HLT* showed a strong decrease of expression (time-point I), followed by recovery to the level observed in the control (time-point II), and then, another slight decrease of expression (time-point III). *LaCAO* expression was significantly inhibited at all stages of infection (time-points I–III). However, susceptible cultivars showed a stronger decrease in expression in relation to the control. Log_2_-transformed mean values of gene expression in individual cultivars and their standard errors are given in Table S7.

### 2.5. QA Gene Expression Changes in response to Colletotrichum lupini Infection in Sweet and Bitter Cultivars

A significant interaction (*p* < 0.01) between the effect of plant bitterness/sweetness and time-point of infection was revealed in the expression of four QA genes; namely, *CCR*, *LaAT*, *LaCAO*, and *LDC* (Table 7). The expression of *CCR*, *DHDPS*, *LaAT*, and *LDC* genes were significantly different between the two groups of cultivars (bitter/sweet), while the expression of *CCR*, *DHDPS*, *HMT/HLT*, and *LaCAO* changed significantly at different infection time-points (Table 7). 

**Figure 5:**
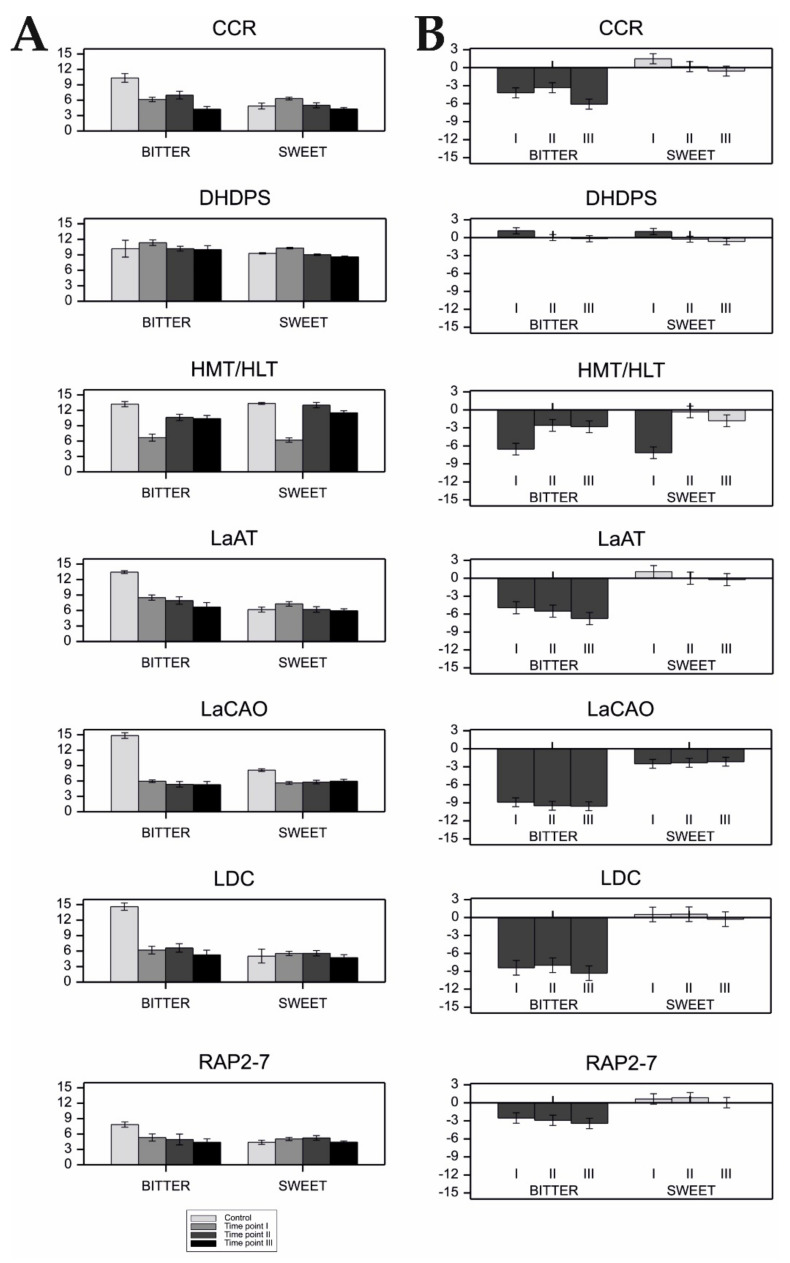
Expression patterns of alkaloid (QA) genes measured during *Colletotrichum lupini* infection in two groups of narrow-leafed lupin cultivars with contrasting seed alkaloid content. Plants with high alkaloid content are referred to as ‘bitter’ (two cultivars) and plants with low alkaloid content are referred to as ‘sweet’ (six cultivars). Full names of genes are listed in Table 1. (**A**) QA gene expression measured in control plants and at three time-points during *Colletotrichum lupini* infection (48 h, 72 h, and 120 h after inoculation). Log_2_-transformed mean values and standard errors are depicted. (**B**) FC values for QA gene expression at consecutive time-points of infection (I–III) in comparison to the control. Dark grey bars indicate statistically different expression (*p* < 0.05). Bars above the x-axis represent increased expression and those below the x-axis represent decreased expression after infection. FC of expression is shown in log_2_ scale.

With the exception of *DHDPS*, the expression of all QA genes significantly decreased in bitter cultivars after infection, remaining suppressed at all further stages of infection (Figure 5). The expression of *DHDPS*, which showed a similar expression pattern in both bitter and sweet cultivars, was increased at time-point I but returned to its previous level at time-points II and III. In sweet cultivars, the expression of *LDC*, *LaAT*, *RAP2-7*, and *CCR* did not change significantly after infection, while the expression of *LaCAO* was decreased at all time-points of the analysis, as compared to the starting levels. Moreover, *HMT/HLT* expression was inhibited at time-point I in sweet cultivars, but returned to the level noted prior to infection afterwards. Log_2_-transformed mean values of gene expression in individual cultivars and their standard errors are given in Table S7.

## 3. Discussion

### 3.1. NLL Alkaloid Biosynthesis Is Conducted Unequally Across Aerial Organs 

Presented results on QA-related genes expression in the individual plant organs of sweet and bitter NLL cultivar strongly support the notion that the vast majority of QA synthesis occurs in the green organs of NLL, which corroborates recent reports investigating QA biosynthesis in this lupin species [16,23,24]. The observed differences in expression levels among organs further imply that their contribution to the TAC is not equal (Figure 1B,C). However, we also demonstrated that the gene expression patterns observed so far in the investigated organs did not fully correspond to their alkaloid contents. This is particularly evident when comparing the moderate QA content in the aerial parts of the bitter plant (especially leaves and stems) to the seeds, which were characterized as having the lowest expression level of the QA genes and the highest QA content (Figure 1B,C and Figure 3, Table S3) [23,24]. Taken together, the results of the present study suggest that a vast majority of NLL alkaloids are transported to the seeds from the sites of their synthesis in the green organs of the plant [12,14,23].

Current observations suggest that the reactions leading to the synthesis of core QAs (e.g., lupanine in NLL) might be interconnected with more broadly distributed subcellular structures, such as chloroplasts, which are known to be a site of lysine biosynthesis and are the key site of *LDC* activity [15,33]. However, it is possible that other, yet unknown genes of the QA synthesis pathway might exhibit more organ-specific expression patterns. For example, in *Nicotiana tabacum*, nicotine N-demethylase of the nornicotine biosynthesis pathway turned out to be expressed exclusively in tobacco ovary [34].

Although we revealed mutual correlations (*p* < 0.01) between *RAP2-7*, *LaCAO*, *LaAT*, *LDC*, and *CCR* gene expression, we also showed that the relationships between *LDC* and other QA genes were not always linear. The further exclusion of roots from the analysis considerably increased the correlation coefficients between the expression of *LDC* and that of other genes. Thus, it could be concluded that the expression of *LDC* was independently regulated in aerial organs and roots. Moreover, we also observed high *LDC* expression in NLL roots of both sweet and bitter cultivars (Figure 1B,C); similar results were previously reported for Oskar [16]. Given that other QA genes were barely expressed in the roots, it is likely that *LDC* functions in other metabolic pathways, possibly in polyamine synthesis; specifically cadaverine. This polyamine is known to play an important role in plant growth, induction of morphological changes, metabolism, and signaling [35,36,37,38]. Cadaverine is known to specifically regulate root growth, increasing lateral branching [36,38], and mediating the stress response by inducing spermine accumulation [39]. A grafting experiment carried out by Lee et al. [14], demonstrated that roots might contribute to the alkaloid synthesis pathway, although at a much lower level than leaves. Given the current results, one possible interpretation is the import of cadaverine from the roots up to the aerial plant organs. However, conclusive resolution of this issue requires follow-up studies. Moreover, the observed *LDC* expression in roots might also imply crosstalk between alkaloid-linked cadaverine synthesis and root nodulation. Polyamines are potential regulators of nutrient exchange in root nodules, which can affect membrane transport, leading to a reduced nitrogen supply to the plant [37,40,41].

### 3.2. Complexity of Influence of RAP2-7 on QA Biosynthesis

Our previous studies showed that APETALA2/ERF RAP2-7 TF, a strong candidate for a master regulator of the QA synthesis pathway in NLL, was upregulated in the leaves of bitter phenotypes [28]. Other known QA genes were found to be located elsewhere than the *iucundus* linkage group/pseudochromosome [27], indirectly supporting a manner of regulation typical for TFs. Representatives of the APETALA2/ERF family are widely reported to play significant roles in the regulation of secondary metabolite pathways in other plant species [42,43,44,45,46,47,48].

In light of previous results for leaves, we hypothesized that *RAP2-7* would also be relatively highly expressed in other aerial organs of the bitter cultivar, which was indeed observed in the leaves, stems, and pods of the Oskar cultivar. Similar expression patterns were observed for *LDC*, *LaCAO*, *LaAT*, and *CCR* genes (Figure 1B) across the organs of Oskar, and statistical analysis indicated the mutual regulation of these genes (Table 4, Table S5). These results support the role of *RAP2-7* as a vital regulatory gene of alkaloid biosynthesis/accumulation in narrow-leafed lupin.

Nonetheless, in bitter vs. sweet FC analysis, significant differences in *RAP2-7* expression were identified only in leaves I and II (Figure 1A). Therefore, the concluded upregulation of *RAP2-7* in leaves I and II of Oskar appeared to be a result of the decreased *RAP2-7* expression in Regent, which was noticeable when compared to its expression level in the other organs of Regent (Figure 1A,C). At the same time, the expression levels of other QA genes in the aerial organs of the sweet cultivar were maintained at a relatively low level (Figure 1A,C), and no correlations were detected between those genes and *RAP2-7* (Table S4). Considering the causes underlying the absence of QA gene expression in the organs of the sweet cultivar, which simultaneously exhibited relatively high levels of *RAP2-7*, the evidence pointed to an inactivating change (or the involvement of additional regulatory factors). Still, a conservative and likely explanation is that a previously identified *RAP2-7* non-synonymous substitution of the amino acid serine (sweet allele) into arginine (bitter allele) (i.e., AGT/CGT, S196R) plays a critical and possibly disruptive role, given the different physicochemical properties of both amino acids. Indeed, involvement of this point mutation in conferring the bitter phenotype is supported by observations when it was exploited for the development of a molecular marker, whereby there was full agreement between marker segregation and seed alkaloid content in an NLL collection (including Oskar and Regent) [28]. Such a strong correlation suggests that the identified SNP is located at a critical site for protein function (i.e., the AP2 domain), possibly mediating protein binding with DNA [49]. There, the substitution of a non-basic serine in the sweet *RAP2-7* allele could weaken RAP2-7 binding, leading to abolished transcriptional induction of the subsequent stages of the QA pathway, and eventually, a marked decline in QA synthesis (i.e., the sweet phenotype with low alkaloid content). Notably, an analogous mechanism was reported for almond (*Prunus amygdalus* L.), where a leucine to phenylalanine substitution in bHLH2 TF prevented the transcription of the two cytochrome P450 genes, which are crucial in amygdaline biosynthesis, thus resulting in desirable sweet kernel genotypes [50]. A different example among AP2/ERF factors concerns the mutation of a cold-induced *BnaERF-B3-hy15* gene in *Brassica napus*. In this case, a mutant protein in which glycine was substituted with arginine in the AP2 domain was found to bind to DNA much stronger than the wild-type protein [51,52]. As a result, the cold-exposed mutant plants showed much stronger upregulation of genes related to cold response than in the wild-type control [53].

Moreover, a follow-up inquiry into the mechanisms that cause decreased expression of *RAP2-7* in sweet leaves (in comparison to its bitter counterparts) might shed new light on the complexity of QA synthesis regulation in NLL. For example, phylogenetic studies on *RAP2-7* revealed that it is related to *Arabidopsis* genes that act as repressors of flowering (i.e., *TOE2*, *SMZ*, and *SNZ*), which were found to be regulated by miR172 [27,54,55,56]. It is possible that the same miRNA family also regulates the expression of *RAP2-7*. However, the most common function of miR172 is translational repression, leading to decreased abundance of the protein but not the mRNA of the target [54,55,57]; this does not explain the results of this study. Other mechanisms of TF-based regulation cannot yet be excluded, such as auto- and crossregulation (mechanisms which are well-recognized in the alkaloid synthesis of *Catharanthus roseus* [58]), epigenetic modifications, the presence of protein domains or partners determining post-translational regulation, or the ubiquitination processes [59].

Follow-up studies on *RAP2-7* expression profiles incorporating a greater number of genotypes are also needed to validate the current data determined for the two NLL genotypes, as well as to provide further details regarding the QA pathway regulation.

### 3.3. Detailed Investigation of DHDPS and CCR as Candidate Genes Connected to QA Synthesis

The *DHDPS* and *CCR* genes, previously selected as QA candidate genes [27], were further studied in the present research. In the bitter cultivar, the *CCR* gene displayed a similar pattern of regulation as other QA-related genes (i.e., *LDC*, *LaCAO*, *LaAT*, and *RAP2-7*; Figure 1A,B) and their patterns of expression were strongly correlated (Table 4, Table S5). These results suggest that *CCR* is regulated in a similar manner to other genes in the QA pathway, and hence, might be involved in the synthesis of derived compounds or conjugates. Pertinently, the homologs of *CCR* are typically involved in the reduction of cinnamoyl-CoA esters into their corresponding hydroxycinnamaldehydes, which is the initial step in the monolignol pathway for lignin biosynthesis [60,61]. Multiple homologs of *CCR* genes are reported to play different roles, even in the same plant species, including participation in defense-related processes and in flavonoid biosynthesis pathways [62]. The latter is possibly indicative of crosstalk between different metabolic pathways.

The lack of correlation between the expression of the *DHDPS* gene and other QA genes, combined with the stable expression of *DHDPS* across all organs, substantiates that it is not directly involved in QA synthesis. The 4-hydroxy-tetrahydrodipicolinate synthase enzyme might be involved in lysine biosynthesis [27]; therefore, its orchestration might be primarily mediated by other regulatory pathways. Interestingly, *DHDPS* in NLL probably exists in the form of two alleles, differentiated by a frameshift-causing deletion of one guanine [27]. If this frameshift was responsible for a non-functional protein in a sweet mutant allele, it could be correlated with lower lysine content, which was indeed reported in some studies [15]. In such a case, the decrease in available substrates for alkaloid synthesis would additionally contribute to lower TAC values.

### 3.4. QAs Possibly Do Not Act as an Additional Line of Defense against Lupin Anthracnose; Possible Role of Plant Polyamines

It was demonstrated that environmental factors might have a significant impact on seed alkaloid content; given their toxicity, the TAC in seeds must be maintained within a safety threshold of 0.02% per dry weight [21,63,64]. Within this study, we sought to find a connection between QA gene expression and plant resistance to *Colletotrichum lupini* (anthracnose), which up until now, were studied separately [5,31,32]. Our results implied that resistance and alkaloid synthesis are independent, which is beneficial from a consumer/food security point of view. We observed that the expression of most tested QA genes either decreased outright or did not change significantly after infection, regardless of cultivar susceptibility (Table 6, Figure 4). This suggests that while QA biosynthesis is influenced by the biotic stress of anthracnose infection, the alkaloids themselves likely do not constitute an active defense system and are regulated as part of the tangential stress response. 

On the other hand, the expression of *LDC*, which is responsible for the decarboxylation of lysine into cadaverine [15,22,25], was found to be downregulated in susceptible but not resistant lines (Figure 4). Therefore, a valid supposition is that polyamines, rather than QAs, might be involved in conferring a degree of plant resistance to anthracnose in NLL. Previous studies on the contribution of polyamines to plant defense responses showed divergent results [38,65]. Whether the results of this study result from the generally better survival rate of the available resistant genotypes in stress conditions or the involvement of cadaverine in the defense response should be clarified in the course of further investigation.

## 4. Materials and Methods 

### 4.1. Plant Material Grown in a Field Experiment to Study the Association Between QA Gene Expression Profiles and Alkaloid Content/Composition

The analyses of gene expression, in conjunction with measurements of alkaloid content, were carried out using two Polish NLL cultivars characterized by their contrasting content of quinolizidine alkaloids (QAs) in dry seeds. These were the sweet (low-alkaloid) cultivar, Regent (Catalogue No. 96245), and the bitter (high-alkaloid) cultivar, Oskar (Catalogue No. 96247), the seeds of which were retrieved from the Polish *Lupinus* Genebank (Poznan Plant Breeders Ltd., Wiatrowo Branch, Poland). Ten to fifteen seeds of each cultivar were sown each year in a field experiment in Wiatrowo, Poland. A natural vernalization process was followed by standard agricultural treatments—mechanical weed control treatment, and fertilizers P_2_O_5_ (60 kg/h) and K_2_O (90 kg/h), during plant growth and development. The field experiment was conducted over four vegetative seasons—2015, 2017, 2018, and 2019.

Plant material was collected yearly for all three phenological phases (i.e., flowering, pod setting, and pod maturing). A total of 13 different organs were sampled during the experiments (Table 2). For a detailed list of plant organs collected and analyzed each year see Tables S1 and S2. Three plants per cultivar, each representing a single biological replicate, were chosen for qPCR and GC analyses. Samples for expression analyses were kept frozen upon collection, until further analysis. The corresponding samples harvested for GC analysis were left to dry. 

### 4.2. Plant Material Grown in a Greenhouse Experiment to Study Expression Changes of QA Genes in Relation to Colletotrichum lupini Infection

Eight NLL cultivars characterized with different susceptibilities to *Colletotrichum lupini* infection and contrasting seed alkaloid content were grown in a greenhouse experiment. The cultivars were contrasted by disease resistance into four cultivars mildly affected by the disease, referred to as ‘resistant’ (Regent, Roland, Sonate, and Tanjil), vs. the four cultivars severely affected by the disease, referred to as ‘susceptible’ (Ernani, Graf, Oskar, and Wersal). The cultivars were then categorized according to alkaloid content, as high seed alkaloid content cultivars (Ernani and Oskar) and low seed alkaloid content cultivars (Graf, Regent, Roland, Sonate, Tanjil, and Wersal). The resistant and susceptible cultivars were recommended by breeders based on their field observations over the years. The seeds were retrieved from the Polish *Lupinus* Genebank (Poznan Plant Breeders Ltd., Wiatrowo Branch, Poland) and Plant Breeding Smolice Ltd., Co. Three seeds were sown per pot (soil and sand mixture; 1:1), serving as one biological replicate. Three biological replicates (pots) were grown and further used for samples collection.

Seedling were grown for 14 days in controlled environment greenhouse (22 °C day/18 °C night, 16 h photoperiod, air humidity ~60–65%), and were manually watered once a week (200 mL). After two weeks, the seedlings were sprayed with a suspension of conidial spores of *Colletotrichum lupini* (2 × 10^6^ conidia per mL) and incubated in darkness for 48 h (25 °C, air humidity ~100%). In the subsequent stage, the growing conditions were set as follows—temperature 22 °C/day and 19–20 °C/night, air humidity ~60–65%. Plant leaves for qPCR analyses were collected, in triplicate, 48 h, 72 h, and 120 h after inoculation with the pathogen (designated as time-point I, II, and III, respectively); leaves collected before inoculation served as controls.

### 4.3. RNA Extraction and Quality Control

RNA was extracted using an SV Total RNA Isolation System Kit (Promega, Madison, WI, USA) from 15–50 mg (leaves and green seeds) or 100–120 mg (remaining organs) of frozen and ground material. RNA purity and concentration were measured with a NanoDrop^®^ Spectrophotometer ND-1000 (Thermo Fisher Scientific, Waltham, MA, USA). The integrity of RNA samples was visualized on non-denaturing 1% agarose gel (90 mV voltage, 30 min); samples with clear 28S and 18S rRNA bands were used for reverse-transcribing into cDNA.

### 4.4. Reverse Transcription 

Reverse transcription was performed with 1000 ng RNA, using a Transcriptor First Strand cDNA Synthesis Kit (Roche, Mannheim, Germany), according to the manufacturer’s protocol, with anchored oligo(dT)18 primer. Two technical replicates were prepared for each sample and subsequently tested for the presence of genomic DNA (gDNA) contamination. For this purpose, conventional PCR (GoTaq^®^ G2 Hot Start DNA polymerase; Promega, Madison, WI, USA) was carried out, with a primer pair designed against two different exons of an *ATP synthase* (*ATPsyn*; LOC109361517) gene sequence (primer sequences: F: AGTATGCTGTTCCTGTTCGTCA; R: ATGGTGATCTTCTCCTTCTTTAG). Two non-contaminated technical replicates of cDNA were mixed and 10-fold or 2-fold samples dilutions were used as template for the evaluation of gene expression. A mixture of non-diluted cDNA (5 µL of each sample) was prepared and used as a template for optimizing qPCR thermal conditions/efficiency, performed separately for the field and greenhouse experiment samples.

### 4.5. Target Genes in Gene Expression Studies and Experimental Design of qPCR Assay

A total of seven genes with known or putative involvement in alkaloid biosynthesis/accumulation selected on the basis of our previous transcriptome-derived investigation [27] were examined within the current study (Table 1). qPCR analyses were conducted using a LightCycler^®^ 480 II instrument and dedicated software (Roche, Mannheim, Germany). Assays were performed with aid of LightCycler^®^ 480 Probes Master (Roche, Mannheim, Germany) and TaqMan probes (Genomed S.A., Poland) labeled with 5′6-FAM and 3′BHQ-1, following the manufacturer’s protocols (reaction volume 10 µL). Primer pairs and probes of target and reference genes were mostly taken from [27] and were designed on the basis of gDNA of 83A:476 (low-alkaloid) and P27255 (high-alkaloid) accessions (Table S8). Three biological and two technical replicates of each sample, as well as a negative control, were included in each assay.

Reaction efficiency was determined for each gene, using a standard curve derived from a pooled cDNA mixture (5–7 serial, 2-fold dilution; three technical replicates) and the 2nd Derivative Max method. PCR amplification efficiencies of the reference genes ranged from 0.94 to 0.97, while those of the tested target genes ranged from 0.94 to 1.00 (Table S8).

To calculate changes in target gene expression, the expression values of individual samples were normalized to the reference genes and subjected to efficiency corrections (these values are referred to as CP_NORM observations) [66].

### 4.6. Determination of Reference Genes Expression Stability 

Nine candidate reference genes [27] were evaluated for their expression stability in a current experimental design (Table S8). Expression stability of these genes was analyzed using the NormFinder and geNorm algorithms, incorporated into the GenEx6 ver. 6 package (Multid Analyses AB, Göteborg, Sweden) as well as the BestKeeper software [67]. Assessment of the most stable reference genes was conducted separately for two subsets of samples: (1) for expression analyses across different plant organs; and (2) for expression analyses after *Colletotrichum* infection, taking into account different time-points of infection. Eventually, the three most stable reference genes for each subset of samples were used to normalize the expression of QA-related genes. For the first subset of samples these were—alcohol dehydrogenase class-3 (*ADH3*) gene, elongation factor 1-beta 2-like (*ELFB*) gene, and tubulin alpha-5 chain (*TUBA5*) gene. For the second subset selected, the reference genes were—*ADH3*, *ELF1B*, and *TUB5* (Control); actin-2-like (*ACT2/7*), *ADH3*, and *TUB5* (Time-point I); glucose-6-phosphate 1-dehydrogenase (*G6PD*), *ACT2/7* and *TUB5* (Time-point II); *ACT2/7*, *ADH3*, and *TUB5* (Time-point III).

### 4.7. Analysis of QA Qualitative and Quantitative Content in Different Lupin Organs by the GC Method

The analysis of alkaloid profiles was carried out using the gas chromatography (GC) assay (GC-2014, Shimadzu, Kyoto, Japan). A detailed description of the alkaloid extraction method, including the technical details of instrument settings, were previously described [68]. Due to the high amount of plant material required for extraction (ca. 500 mg of dry tissue per one technical replicate), each plant organ was analyzed as one representative sample pooled from three biological replicates. Consequently, various types of certain organs (i.e., lateral and main parts of roots and stems) were also combined. Each sample was analyzed using two technical replicates. Quantitative analysis was carried out with the aid of a linear calibration curve made for lupanine using caffeine as an internal standard. Total alkaloid content was evaluated as the percentage of the sum of alkaloids present in the analyzed sample (% of sample dry weight). The relative abundance of each alkaloid was assessed as the percentage of the total alkaloid content in a particular plant organ.

### 4.8. Statistical Analysis

The observations of gene expression were transformed using the formula log_2_(CP_NORM × 10^6^). Observations of alkaloid levels were transformed using the formula log_10_((x + 0.01) × 10^2^). Analysis of variance was performed in a mixed linear model containing the fixed effects of plant organs, cultivars, groups of cultivars, or time-points, and their interactions, as well as the random effects of years (according to the needs of analysis for various experiments and traits). Mean total alkaloid content in organs was obtained by Best Linear Unbiased Prediction in the mixed linear model. Post-hoc comparisons and groupings of mean values of observed traits were made using Fisher’s protected LSD test. The significance of Pearson’s correlation coefficients was assessed using the test, based on the *t*-distribution. All above analyses and visualization of the results were carried out using the Genstat ver. 19 software [69].

## 5. Conclusions

At present, there remain gaps in our knowledge about the precise regulation of QA synthesis in NLL. Within the framework of the current investigation, we identified the upregulation of *RAP2-7* and other QA genes across the aerial organs of a bitter cultivar, in parallel with significant correlations of transcript expression, thus supporting the role of *RAP2-7* as a vital regulatory gene of alkaloid biosynthesis/accumulation in narrow-leafed lupin. On the other hand, the unexpected expression patterns of *RAP2-7* in the organs of a sweet cultivar implied that other aspects of QA biosynthesis regulation might be involved. Undoubtedly, in the aerial organs (i.e., stems and young pods) of the sweet cultivar, despite relatively high *RAP2-7* expression, the expression of other genes of the QA synthesis pathway were maintained at a much lower level when compared with those of the corresponding organs of the bitter cultivar. Therefore, further research is required to resolve the question of how the RAP2-7 S196R substitution affects its function in the sweet cultivar.

We also demonstrated that at least several steps of alkaloid synthesis (converted by the products of investigated genes) might occur independently in organs other than leaves, thus supporting the role of *RAP2-7* as a common regulator of the pathway. Follow-up studies on the mechanisms of its action and effect on regulating expression are necessary to obtain a clear understanding of this important metabolic pathway.

Moreover, in the limited context of the eight NLL cultivars characterized by their differing resistance/susceptibility to *Colletotrichum lupini* infection and contrasting seed alkaloid content, we also demonstrated that the alkaloid content in NLL seeds was seemingly unperturbed after anthracnose infection, even though other lysine-derived metabolites, such as polyamines, might play roles in the defense against this fungal disease. Direct investigation of the contribution of lupin polyamines to the plant defense response might cast new light on this important issue.

## Figures and Tables

**Table 1:** Structural and candidate genes involved in quinolizidine alkaloids biosynthesis and accumulation in narrow-leafed lupin that were assayed by quantitative PCR (qPCR).

Target Gene	Description	References
*LDC*	lysine decarboxylase	Bunsupa, et al. [15]
*LaAT*	acyltransferase-like gene	Bunsupa, et al. [8]
*LaCAO*	copper amine oxidase	Yang, et al. [16]
*HMT/HLT*	acyltransferase tigloyl-CoA:(−)-13α-hydroxymultiflorine/(+)-13α-hydroxylupanine O-tigloyltransferase	Suzuki, et al. [13]; Okada, et al. [20]
*RAP2-7*	APETALA2/ethylene response transcription factor	Kroc, et al. [27]
*DHDPS*	4-hydroxy-tetrahydrodipicolinate synthase	Kroc, et al. [27]
*CCR*	cinnamoyl-reductase 2	Kroc, et al. [27]

**Table 2:** Plant samples collected for two narrow-leafed lupin cultivars (Oskar and Regent), incorporated into gene expression qPCR analyses, as well as for evaluation of alkaloid content by gas chromatography (GC) assay. Detailed list of organs collected and analyzed each year is shown in Tables S1 and S2.

Plant Phenological Phase	Organ	Description	Gene Expression (qPCR)	Alkaloid Content (GC)
Flowering (approximately 60 DAS **^1^**)	Leaves I	The uppermost leaves, not fully expanded	+ **^2^**	+
	Main stem	Plant stem, emerging directly from the roots	+	+
	Lateral stems	Lateral branches, emerging from the main stem	+	
	Flowers	Flower buds or open flowers from primary or secondary inflorescences	+	+
	Main root	Primary root	+	+
	Lateral roots	Lateral roots emerging from the primary root	+	
Pod setting (approx. 70 DAS^1^)	Leaves II	Fully expanded uppermost leaves	+	+
	Young pods	Immature green pods, 2 cm in length, containing seeds	+	+
Pod maturing (approx. 85 DAS^1^)	Leaves III	Uppermost, not fully wilted leaves	+	+
	Green pods	Pod walls separated from matured green pods, 3 to 5 cm in length	+	+
	Green seeds	Green ripened seeds collected from mature green pods	+	+
	Dry seeds	Dried mature seeds		+
	Dry pods	Dried mature pod walls		+

**^1^** Days after sowing; **^2^** analyses performed for a given plant organ.

**Table 3:** Summary of ANOVA analysis for testing the effect of plant organ, cultivar, and their interaction on the expression of alkaloid genes (qPCR) in two narrow-leafed lupin cultivars—Oskar (high-alkaloid, bitter) and Regent (low-alkaloid, sweet). Effects were considered significant for *p* < 0.01 (marked in bold). Full names of genes are listed in Table 1.

No.	Gene	*p*-Value for		
		Organ	Cultivar	O × C Interaction
1	*CCR*	**<0.001**	**<0.001**	**<0.001**
2	*DHDPS*	**<0.001**	**<0.001**	0.037
3	*HMT/HLT*	**<0.001**	0.541	**0.002**
4	*LaAT*	**<0.001**	**<0.001**	**<0.001**
5	*LaCAO*	**<0.001**	**<0.001**	**<0.001**
6	*LDC*	**<0.001**	**<0.001**	**<0.001**
7	*RAP2-7*	**<0.001**	**0.001**	**<0.001**

**Table 4:** Pearson correlation coefficients (significant at *p* < 0.01; *n* = 81) between the expression values of alkaloid-related genes, measured for all organs of the bitter narrow-leafed lupin cultivar Oskar using qPCR assay. Full names of genes are listed in Table 1.

	Pearson Correlation Coefficients						
Gene	*CCR*	*DHDPS*	*HMT/HLT*	*LaAT*	*LaCAO*	*LDC*	*RAP2-7*
*CCR*	1						
*DHDPS*	0.39	1					
*HMT/HLT*	0.57	n.s	1				
*LaAT*	0.91	0.39	0.64	1			
*LaCAO*	0.87	n.s	0.58	0.92	1		
*LDC*	0.78	0.29	0.46	0.83	0.74	1	
*RAP2-7*	0.87	0.36	0.68	0.82	0.74	0.71	1

n.s.—lack of significant correlation.

**Table 5:** Summary of ANOVA analysis testing the effects of plant organ, plant cultivar, and their interaction on the relative abundances of particular alkaloids in two narrow-leafed lupin cultivars—Oskar (bitter phenotype) and Regent (sweet phenotype). Effects were considered significant for *p* < 0.01 (marked in bold).

No.	Alkaloid	*p*-Value		
		Organ (O)	Cultivar (C)	O × C interaction
1	10,17-dioxosparteine	0.424	0.034	0.365
2	11,12-didehydrolupanine	0.651	**<0.001**	0.626
3	11,12-secodidehydromultiflorine	0.385	0.127	0.863
4	13-angelolyoxylupanine	0.987	0.392	0.967
5	13-angelolyoxymultiflorine	0.189	**<0.001**	0.189
6	13-benzoyloxylupanine	**0.001**	0.296	0.020
7	13-hydroxylupanine	0.619	0.078	0.970
8	13-hydroxymultiflorine	0.317	**<0.001**	0.072
9	13-tigloyloxymultiflorine	0.065	**0.003**	0.158
10	5,6-didehydrolupanine	0.891	**0.001**	0.966
11	albine	0.895	0.114	0.903
12	ammodendrine	0.386	**0.010**	0.214
13	angustifoline	0.041	**<0.001**	0.285
14	isolupanine	0.897	**<0.001**	0.584
15	lupaninie	0.429	**0.001**	0.786
16	multiflorine	0.314	0.977	0.852
17	oxolupanine	0.181	**<0.001**	0.207
18	sparteine	0.839	0.578	0.815

**Table 6:** Summary of ANOVA for two groups of narrow-leafed lupin cultivars with different infection resistance levels: plants mildly affected by the disease are referred to as ‘resistant’ (four cultivars), while plants severely affected by the disease are referred to as ‘susceptible’ (four cultivars). Expression was analyzed by qPCR assay. Effects were considered significant for *p* < 0.01 (marked in bold). Full names of genes are listed in Table 1.

No.	Gene	*p*-Value		
		Groups of Cultivars (Resistant, Susceptible) (R/S)	Time Points (T)	R/S × T Interaction
1	*CCR*	0.043	**<0.001**	0.094
2	*DHDPS*	**0.001**	**<0.001**	0.735
3	*HMT/HLT*	0.542	**<0.001**	0.646
4	*LaAT*	**0.007**	0.030	0.034
5	*LaCAO*	0.207	**<0.001**	0.010
6	*LDC*	**< 0.001**	0.054	0.012
7	*RAP2-7*	0.288	0.297	0.355

**Table 7:** Summary of ANOVA for two groups of narrow-leafed lupin cultivars with contrasting seed alkaloid content—plants with high alkaloid content are referred to as ‘bitter’ (two cultivars) and plants with low alkaloid content are referred to as ‘sweet’ (six cultivars). Expression was analyzed by qPCR assay. Effects were considered significant for *p* < 0.01 (marked in bold). Full names of genes are listed in Table 1.

No	Gene	*p*-Value		
		Groups of Cultivars (Bitter/Sweet) (B/S)	Time Points (T)	B/S × T Interaction
1	*CCR*	**0.007**	**<0.001**	**<0.001**
2	*DHDPS*	**<0.001**	**<0.001**	0.924
3	*HMT/HLT*	0.042	**<0.001**	0.104
4	*LaAT*	**<0.001**	0.016	**0.003**
5	*LaCAO*	0.198	**<0.001**	**<0.001**
6	*LDC*	**0.005**	0.046	**<0.001**
7	*RAP2-7*	0.416	0.278	0.076

## Supplementary Materials

Supplementary materials can be found at https://www.mdpi.com/1422-0067/22/5/2676/s1.

## Abbreviations

*CCR*	cinnamoyl-reductase 2
*DHDPS*	4-hydroxy-tetrahydrodipicolinate synthase
FC	fold-change
GC	gas chromatography
*HMT/HLT*	acyltransferase tigloyl-CoA:(−)-13α-hydroxymultiflorine/(+)-13α-hydroxylupanine O-tigloyltransferase
*LaCAO*	Lupinus angustifolius copper amine oxidase
*LaAT*	*Lupinus angustifolius* acyltransferase-like
*LDC*	lysine decarboxylase
LSD	least significant difference
NLL	narrow-leafed lupin
QA	quinolizidine alkaloids
qPCR	quantitative polymerase-chain reaction
*RAP2-7*	APETALA2/ethylene response, RAP2-7 transcription factor
SE	standard error
TAC	total alkaloid content
TF	transcription factor
