# Supplementary material for: Expression Profiles of Alkaloid-Related Genes across the Organs of Narrow-Leafed Lupin (Lupinus angustifolius L.) and in Response to Anthracnose Infection
Source: Int J Mol Sci. 2021 Mar 6;22(5):2676. doi: 10.3390/ijms22052676 (PMC7962062; doi:10.3390/ijms22052676)
Supplement: Supplementary file 1 [file ijms-22-02676-s001.zip › Supplementary Table S4.docx]

**Table S4.** Pearson correlation coefficients (significant at P < 0.01; n = 81) between the expression values of alkaloid-related genes measured in the organs of a sweet cultivar, Regent, by quantitative PCR (qPCR) assay. Full names of genes are listed in Table 1.

|  | **Pearson correlation coefficients** | | | | | |  |
| --- | --- | --- | --- | --- | --- | --- | --- |
| **Gene** | *CCR* | *DHDPS* | *HMT/HLT* | *LaAT* | *LaCAO* | *LDC* | *RAP2-7* |
| *CCR* | 1 |  |  |  |  |  |  |
| *DHDPS* | 0.34 | 1 |  |  |  |  |  |
| *HMT/HLT* | n.s. | n.s. | 1 |  |  |  |  |
| *LaAT* | 0.53 | 0.38 | n.s. | 1 |  |  |  |
| *LaCAO* | n.s. | n.s. | 0.28 | n.s. | 1 |  |  |
| *LDC* | n.s. | n.s. | n.s. | 0.29 | n.s. | 1 |  |
| *RAP2-7* | 0.44 | n.s. | 0.48 | 0.50 | 0.28 | n.s. | 1 |

n.s. - correlation not significant
